# RNF8 induces β-catenin-mediated c-Myc expression and promotes colon cancer proliferation

**DOI:** 10.7150/ijbs.44119

**Published:** 2020-05-01

**Authors:** Ling Ren, Tingting Zhou, Yang Wang, Yanmei Wu, Hongde Xu, Jingwei Liu, Xiang Dong, Fei Yi, Qiqiang Guo, Zhuo Wang, Xiaoman Li, Ning Bai, Wendong Guo, Min Guo, Bo Jiang, Xuan Wu, Yanling Feng, Xiaoyu Song, Siyi Zhang, Yue Zhao, Liu Cao, Shuai Han, Chengzhong Xing

**Affiliations:** 1 Department of Anorectal Surgery, the First Affiliated Hospital of China Medical University, Shenyang 110001, RP China; 2 Institute of Translational Medicine, College of Basic Medicine, China Medical University, Shenyang 110122, RP China; 3 Panjin Liaohe Oilfield Gem FLower Hospital, Panjin 7650036, RP China; 4 Department of Cell Biology, Key Laboratory of Cell Biology, Ministry of Public Health, and Key Laboratory of Medical Cell Biology, Ministry of Education, School of Life Sciences, China Medical University, Shenyang 110122, RP China; 5 Department of Neurosurgery, the First Affiliated Hospital of China Medical University, Shenyang 110001, RP China

**Keywords:** RNF8, c-Myc, β-catenin, colon cancer

## Abstract

DNA damage signals transducer RING finger protein 8 (RNF8) is involved in maintaining genomic stability by facilitating the repair of DNA double-strand breaks (DSB) via ubiquitin signaling. By analyzing the TCGA database and colon cancer tissue microarrays, we found that the expression level of RNF8 was positively correlated with that of c-Myc in colon cancer, which were closely associated with poor survival of colon cancer patients. Furthermore, overexpressing and knocking down RNF8 increased and decreased the expression of c-Myc in colon cancer cells, respectively. In addition, RNF8 interacted with β-catenin and facilitated its nuclear translocation by conjugating K63 polyubiquitination on it. These observations suggested a de novo role of RNF8 in promoting the progression of colon cancer by inducing β-catenin-mediated c-Myc expression.

## Introduction

Colorectal cancer (CRC) is the fourth most commonly diagnosed and the second leading cause of cancer mortality worldwide 1. In recent years, new treatments for CRC have been developed, which have improved the overall survival of CRC patients significantly 2-4. Nevertheless, 50% of CRC patients undergo disease recurrence 5. Therefore, it is urgent to unravel the underlying molecular mechanisms of CRC oncogenesis and progression to identify novel therapeutic targets.

Overexpression of oncogenes such as *MYC* is one of the major drivers for the initiation and progression of CRC 6-8. The MYC family regulates cell survival, apoptosis, proliferation, and differentiation 9-10. Deletion of *MYC* rescues the colorectal tumor phenotype of the *Apc*-deficient mouse model 11, suggesting an important role of MYC in colorectal tumorigenesis. As the most oncogenic protein of the MYC family, c-Myc is universally dysregulated in CRC and associated with a poor prognosis of CRC patients 12-14. c-Myc acts as a transcription factor regulating a large number of genes to promote cell proliferation 15-17. The expression of c-Myc is strictly regulated by the Wnt/ β-catenin pathway 18. Dysregulation of this pathway usually leads to upregulation of c-Myc by promoting accumulation and nuclear translocation of β-catenin, which contributes to CRC oncogenesis and progression 6, 19. Thus, exploration of the molecular mechanisms regulating c-Myc expression may shed light on improving therapies for CRC patients.

RING finger protein 8 (RNF8), which belongs to the RING finger E3 ligase family, is an essential factor for transducing DNA double-strand breaks (DSB) signaling 20-22. It has been reported to be highly expressed in breast cancer and positively correlated with the progression of breast cancer by activating the Wnt/β-catenin pathway or enhancing the activity of Twist and estrogen receptor α (ERα) 23-25. These findings suggest an essential role of RNF8 in the progression of cancer. However, the exact function and mechanism of RNF8 in regulating the progression of CRC remain largely unknown.

In this study, we found a positive correlation between RNF8 and c-Myc in colon cancer tissues. Subsequently, our results showed that RNF8 induced c-Myc expression via binding to β-catenin and facilitating its nuclear translocation by conjugating K63 polyubiquitination onto it. Furthermore, knockdown of RNF8 significantly inhibited the growth of colon cancer cells *in vitro* and *in vivo*. These results indicate that RNF8 promotes colon cancer proliferation by upregulating β-catenin-mediated c-Myc expression, and provide a new insight into the mechanism of colon cancer progression.

## Materials and Methods

### Immunohistochemistry

The tissue microarray was dewaxed in xylene and rehydrated with decreasing concentrations of ethanol. Endogenous peroxidase activity was blocked by peroxidase blocker (KIT-9720-A) and the tissue microarray was treated with non-immune goat serum (KIT-9720-B). The tissue microarray was incubated with anti-RNF8 or -c-Myc antibodies, followed a biotin-labeled secondary antibody (KIT-9720-C), avidin-peroxidase reagent (KIT-9720-D), and then diaminobenzidine (Zhongshan biotech).

The staining was assessed by double-blinded evaluation. The staining intensity was scored as 0-3 (0: negative; 1: weak; 2: medium; 3: strong). The percentage of positively stained cells was scored as 1-4: (1: 0%-25%, 2: 26%-50%, 3: 51%-75%, 4: 76%-100%). The staining index (SI=intensity score×percentage) was used to assess the level of staining. SI≥8 was defined as high expression and SI<8 was defined as low expression.

### RNA isolation and quantitative real-time PCR

Total RNA was isolated from cells using Trizol reagent (Thermo Fisher Scientific, 15596026). Reverse transcription was performed using a PrimeScript RT Reagent Kit (TAKARA, RR037A), according to the manufacturer's instructions. The cDNA was quantified by quantitative real-time PCR with SYBR®Premix Ex Taq™ II (TAKARA, RR820A) on Mx3000P instrument (Agilent StrataGene). The sequences of the primers were shown in Table S6. Gene expression was calculated relative to expression of the housekeeping gene *GAPDH* using Stratagene Mx3000P software.

### Nuclear extraction

Cytoplasmic and nuclear fractions were extracted from cells using a Minute™ Cytoplasmic and Nuclear Extraction Kit (Invent Biotechnologies, Shanghai, China), according to the manufacturer's instructions.

### Ubiquitination assay

Colon cancer cells were co-transfected with Flag-RNF8 and ubiquitin plasmids and treated with 20 μM MG132 (Selleck, S2619) for 30 minutes. The cells were lysed in 100 μl lysis buffer (50 mM Tris-HCl, pH 7.5, 0.5 mM EDTA, 1 mM DTT, 1% SDS, and a protease inhibitor) and incubated at 95°C for 5 minutes. Then, the cells were added with 900 μl Tris-HCl buffer (50 mM, pH 7.5) and sonicated. After centrifugation, the cell lysates were subjected to immunoprecipitation.

### Proliferation assays and colony formation

For proliferation assay, 3×10^3^ cells per well were cultured in 96-well plates in triplicate. The cell number was then detected using CCK8 (Bimake, B34304) reagent by measuring the absorbance at 450 nm with a microplate reader (TECAN, Switzerland).

For the colony formation assay, 1×10^3^ cells per well were seeded on 6-well plates in triplicate and maintained in medium containing 10% FBS. After 10 days, the cells were fixed with 4% paraformaldehyde for 15 minutes at room temperature and stained with coomassie brilliant blue (R250) for 15 minutes at room temperature and were imaged.

### Tumor xenografts

Twenty nude mice (BALB/C, 4-weeks-old, female) were purchased from Shenyang Huafukang Biosciences (Shenyang, China). The animal experiments meet the requirements of the Animal Care and Use Committee of China Medical University. The mice were divided into two groups of 10 mice each, and 5×10^6^ HCT116 cells with stable knockdown of RNF8 or control cells were injected subcutaneously. The tumor volume was measured daily using the formula π/6×r_1_^2^×r_2_ (r_1<_r_2_). After 13 days, the mice were sacrificed and the tumors were isolated, photographed, measured, and subjected to immunohistochemistry.

## Results

### RNF8 expression increases in colon cancer and is positively correlated with c-Myc

**Figure 1:**
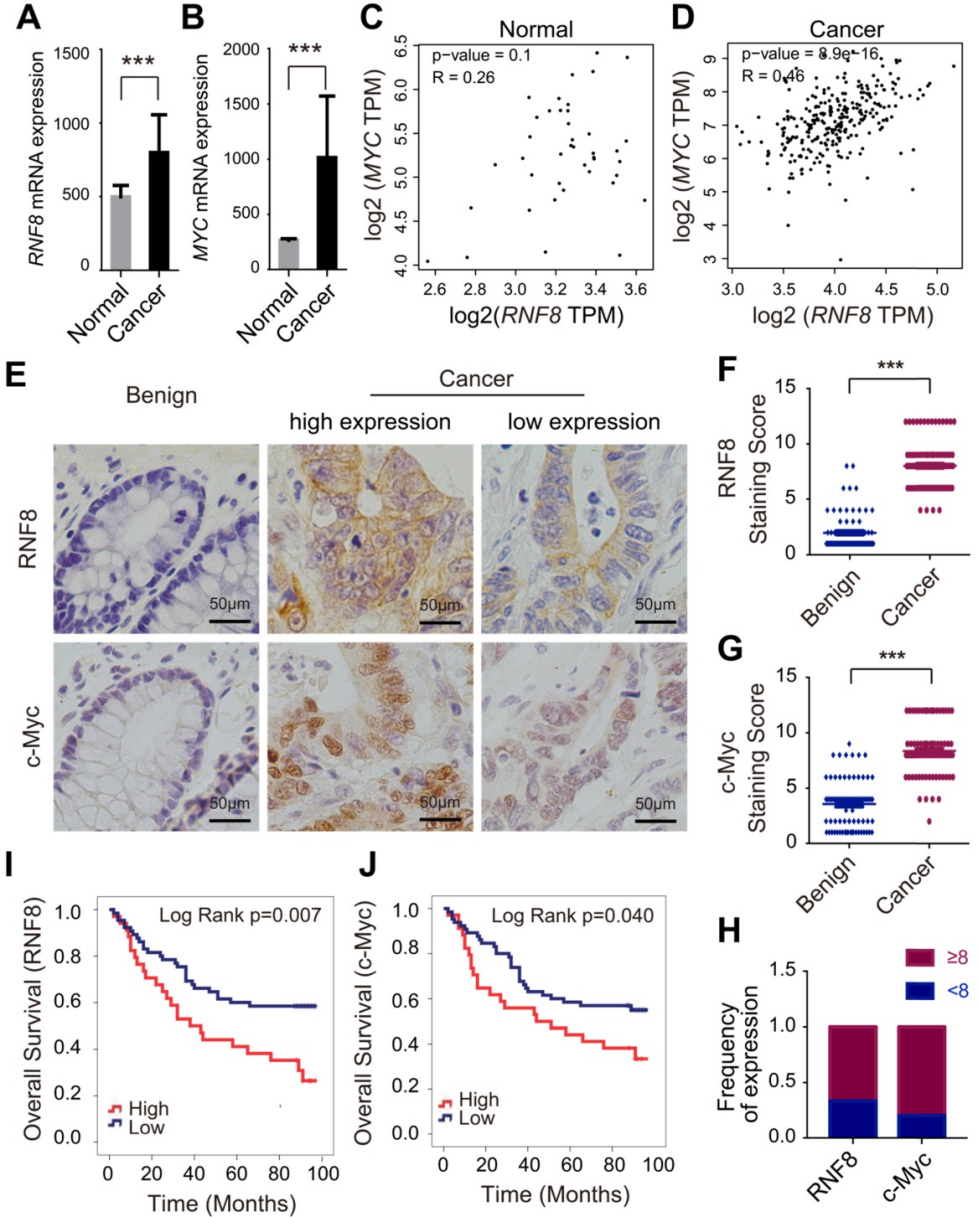
** RNF8 expression increases in colon cancer and is positively correlated with c-Myc expression**. **(A-B)** *RNF8* and *MYC* mRNA levels were high in colon cancer compared with normal controls. Quantitative analysis of *RNF8* and *MYC* mRNA expression in 478 cases of colon cancer compared with 41 normal tissues based on the TCGA database (***p<0.001). **(C-D)** The mRNA level of *RNF8* was positively correlated with that of *MYC* in colon cancer. Scatterplot analysis of the correlation between *RNF8* and *MYC* mRNA expression in normal colon tissue (C) (p=0.1) and colon cancer (D) (p=8.9e-16). **(E)** RNF8 and c-Myc staining levels were higher in colon cancer than in benign tissues. Representative images of RNF8 and c-Myc immunohistochemical staining in colon cancer and benign tissues. Scale bar, 50 μm. **(F-G)** Statistical analysis of RNF8 (F) and c-Myc (G) expression in colon cancer compared with benign tissues (***p<0.001). **(H)** Expression frequency of RNF8 and c-Myc equal to or more than and less than the median of the staining score (score 8) of colon cancer tissues. **(I-J)** High expression of RNF8 and c-Myc was correlated with a poor prognosis of colon cancer patients. Kaplan-Meier analysis of overall survival of colon cancer tissue microarray data with low or high expression of RNF8 (I) or c-Myc (J).

In tissue microarray staining based on The Human Protein Atlas (http://www.proteinatlas.org), six of twelve patients (50%) showed moderate/strong staining of RNF8 in colon cancer. Therefore, we suspected a function of RNF8 in colon cancer progression. To investigate the role of RNF8 in colon cancer, we first analyzed its expression along with that of oncogene *MYC*, which is frequently dysregulated in colon cancer 26, in 478 colon cancer patients and 41 normal controls based on the TCGA database. The results showed that the mRNA level of *RNF8* was elevated in colon cancer tissues compared with normal controls (Figure 1A), as well as that of *MYC* (Figure 1B). Interestingly, according to the data from GEPIA (http://gepia.cancer-pku.cn/index.html) 27 and the analysis from TCGA database, mRNA levels of *RNF8* and *MYC* were positively correlated with each other in colon cancer tissues but not in normal tissues (Figure 1C and 1D, Table S1), indicating that *RNF8* might have a specific relationship with *MYC* in colon cancer.

Next, we performed immunohistochemistry of RNF8 and c-Myc in colon cancer tissue microarrays containing 99 cancer tissues and 78 benign tissues. Consistent with the TCGA data, the protein levels of *RNF8* and *MYC* gene in colon cancer tissues were both significantly higher than those in benign tissues (Figure 1E), as indicated by analysis of the staining scores (Figure 1F and 1G). Furthermore, the expression of RNF8 was positively correlated with that of c-Myc in colon cancer tissues (Figure 1E and 1H, Table S2). To further investigate whether RNF8 was related to the prognosis of colon cancer patients, we performed survival analysis by the Kaplan-Meier curve with the log-rank test in these colon cancer microarray samples. The results showed that, similar to high expression of c-Myc in colon cancer cases, patients with high expression of RNF8 had poor overall survival (Figure 1I and 1J). Cox regression analysis revealed that colon cancer patients with high RNF8 expression had a 2.101-fold increased risk of death compared with patients with low RNF8 expression (HR=2.101, 95% CI=1.210-3.648, p=0.008), and patients with high c-Myc expression had a 1.769-fold increased risk of death compared with low c-Myc expression (HR=1.769, 95% CI=1.015-3.082, p=0.044) (Table S3), suggesting a potential relationship of RNF8 with c-Myc in the poor prognosis of colon cancer patients. The relationships of RNF8 and c-Myc staining with clinicopathological features of colon cancer patients are summarized (Table S4).

These results demonstrated that RNF8 was highly expressed in colon cancer tissues and closely correlated with c-Myc expression. High expression of RNF8 indicated poor survival of colon cancer patients. This suggested that RNF8 participated in colon cancer progression, which was associated with c-Myc.

### RNF8 induces c-Myc expression via β-catenin

**Figure 2:**
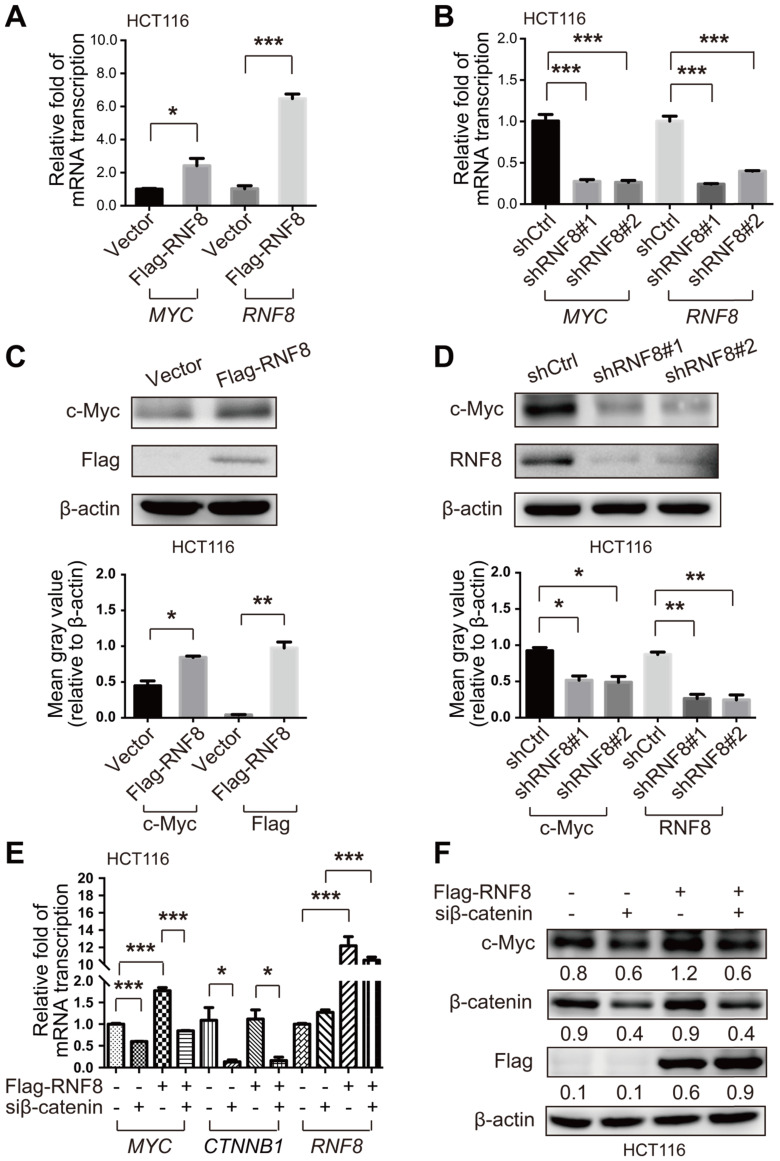
** RNF8 induces c-Myc expression via β-catenin. (A-B)** Overexpression and knockdown of RNF8 increased and decreased *MYC* transcription, respectively. HCT116 cells were transfected with Flag-RNF8 or knocked down RNF8 with specific shRNA. The transcription levels of *MYC* and *RNF8* were represented as the ratio normalized to the transcription level of housekeeping gene *GAPDH*. *p<0.05; ***p<0.001 (Student's *t*-test) compared with the vector and shCtrl controls. **(C-D)** Overexpression and knockdown of RNF8 increased and decreased the c-Myc protein level, respectively. HCT116 cells were overexpressed with Flag-RNF8 or silenced RNF8 with specific shRNA. The protein level of c-Myc was determined by western blotting with the indicated antibodies. Below the western blot images, gray value analyses of protein expression are shown as the mean ± SD, *p<0.05; **p<0.01 (Student's *t*-test) compared with vector and shCtrl controls. **(E)** Silencing of β-catenin compromised the enhancing effect of RNF8 overexpression on *MYC* transcription. HCT116 cells were transfected with Flag-RNF8 with or without β-catenin knockdown using specific siRNA. The mRNA expression of *MYC*, *CTNNB1*, and *RNF8* was represented as the ratio normalized to the transcription level of *GAPDH*. *p<0.05; ***p<0.001 (Student's *t*-test) compared with the vector and siCtrl controls. **(F)** The enhancing effect of RNF8 overexpression on c-Myc expression was dependent on β-catenin. HCT116 cells were cotransfected of Flag-RNF8 with or without siβ-catenin and then the c-Myc protein level was determined by western blotting.

Because previous studies have reported that RNF8 participates in regulation of various genes, which are the targets of Twist and ERα 24-25, we explored whether RNF8 regulated the expression of c-Myc. To begin with, we used the Cancer Cell Line Encyclopedia (CCLE) database 28 to examine RNF8 expression levels in various colon cancer cell lines. We selected HCT116 and SW480 cell lines with relatively high expression of RNF8 and mutant components of the Wnt/β-catenin pathway 29 (Figure S1A). In both HCT116 and SW480 cells, we found that overexpression and knockdown of RNF8 increased and decreased *MYC* gene transcription, respectively (Figure 2A and 2B, Figure S2A and S2B). The silencing efficacy of the shRNA targeting RNF8 in HCT116 and SW480 cells was shown in Figure S1B and S1C, respectively, and we chose the most efficient one for the experiments. Moreover, the protein level of c-Myc was elevated by overexpressing RNF8 and decreased by silencing RNF8 in HCT116 and SW480 cells, respectively (Figure 2C and 2D, Figure S2C and S2D).

β-catenin is an essential factor to activate c-Myc expression 30. Hence, to further explore how RNF8 regulates c-Myc expression, we simultaneously overexpressed RNF8 and silenced β-catenin in HCT116 and SW480 cells, and detected the expression level of c-Myc. The results showed that the inducing effect of RNF8 overexpression on c-Myc expression was dramatically diminished when β-catenin was silenced in HCT116 and SW480 cells (Figure 2E and 2F, Figure S2E and S2F). The knockdown efficacy of the siRNA targeting β-catenin in HCT116 and SW480 cells was determined and the most efficient sequence was chosen for the experiments (Figure S1D and S1E). These results suggested that RNF8 induced c-Myc expression dependent on β-catenin.

### RNF8 facilitates β-catenin nuclear translocation

**Figure 3:**
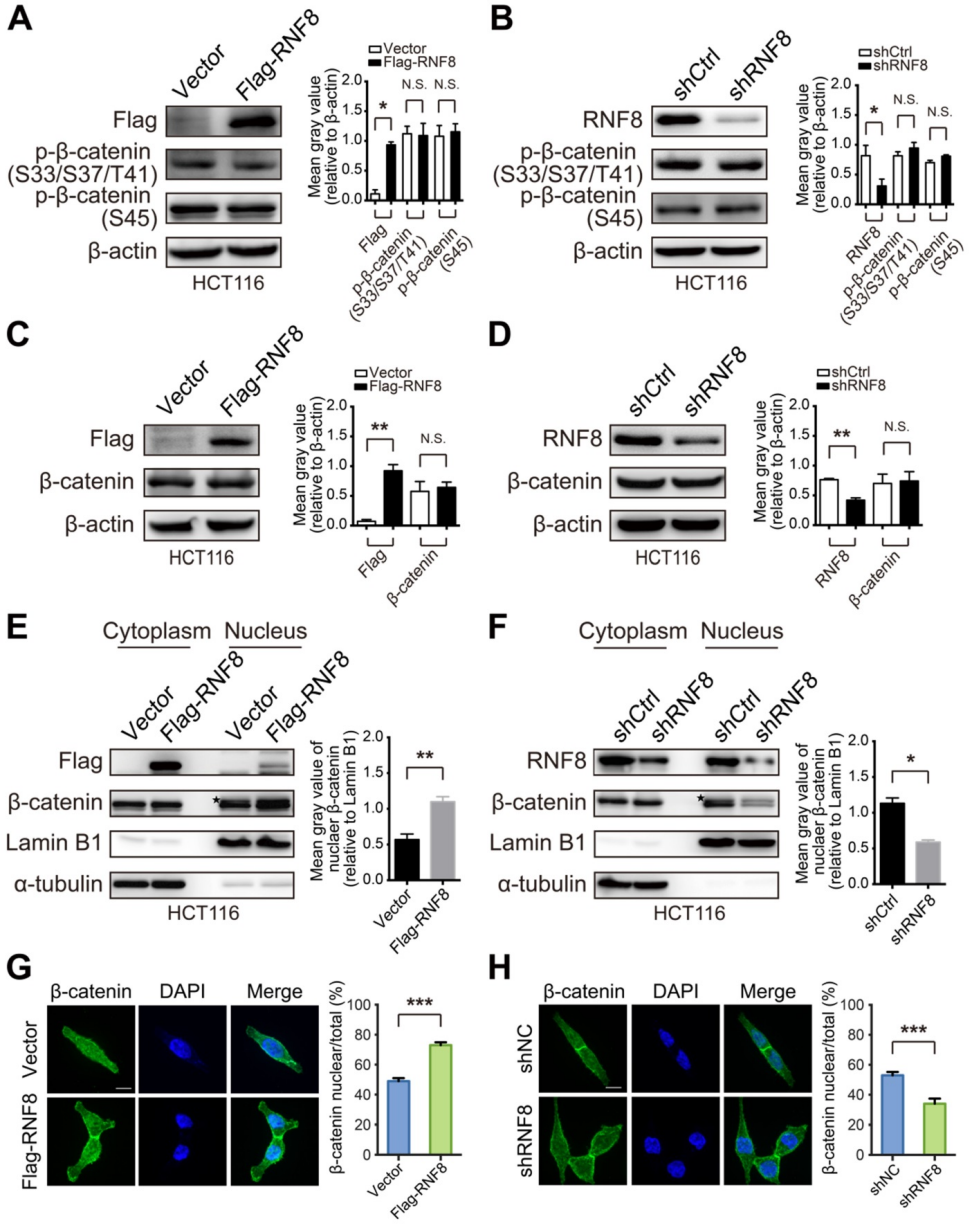
** RNF8 facilitates β-catenin nuclear translocation. (A-B)** Overexpression or knockdown of RNF8 hardly influenced the phosphorylation level of β-catenin. RNF8 was overexpressed or knocked down in HCT116 cells, and phosphorylation of β-catenin at S33/S37/T41 and S45 was detected by western blotting. The gray value analyses are shown on the right, *p<0.05; N.S.=no significant difference (Student's *t*-test) compared with vector and shCtrl controls. **(C-D)** The protein level of β-catenin showed little alteration when RNF8 was overexpressed or knocked down. HCT116 cells were transfected with Flag-RNF8 or underwent knockdown with specific shRNA. Western blotting was performed to measure the protein level of β-catenin in cell lysates. The gray value analyses are shown on the right, **p<0.01; N.S.=no significant difference (Student's *t*-test) compared with the vector and shCtrl controls. **(E-F)** Overexpression and silencing of RNF8 increased and decreased the nuclear translocation of β-catenin, respectively. The protein levels of β-catenin in the cytoplasm and nucleus were determined in HCT116 cells with overexpression or knockdown of RNF8 by a nuclear extraction assay. Asterisk indicates a non-specific band. Gray value analyses of β-catenin in the nuclear fraction are shown at the right side of western blot images, *p<0.05; **p<0.01 (Student's *t*-test) compared with vector and shCtrl controls. **(G-H)** Immunofluorescence analysis of β-catenin nuclear translocation induced by overexpression and inhibited by silencing of RNF8. RNF8 overexpressing and RNF8 knockdown HCT116 cells were fixed and incubated with an anti-β-catenin antibody, followed by an Alex Fluor 488 (green) secondary antibody. Nuclei were counterstained with DAPI (blue). Scale bar, 10 μm. Quantitative analyses of nuclear imported β-catenin relative to total cells are shown on the right. ***p<0.001 (Student's *t*-test) compared with vector and shCtrl controls.

Because our results demonstrated that RNF8 upregulated c-Myc expression via β-catenin, we next investigated the mechanism of this effect. Constitutively activated Wnt/β-catenin pathway is a major driver of colon cancer, and β-catenin accumulation and nuclear translocation are crucial processes in this pathway to induce c-Myc expression leading to colon cancer development 17, 31-34. When the Wnt/β-catenin pathway is activated, dephosphorylation of β-catenin at serine (S) and threonine (T) residues S33/37 and T41 by GSK3β and S45 by CK1 is critical for transcriptional activation of Wnt target genes 35. Thus, we examined whether RNF8 influenced the phosphorylation level of β-catenin at these sites. However, overexpression or knockdown of RNF8 in HCT116 and SW480 cells hardly affected the phosphorylation of β-catenin at S33/37/T41 and S45 sites (Figure 3A and 3B, Figure S3A and S2B), nor the protein level of β-catenin (Figure 3C and 3D, Figure S3C and S3D). These results were consistent with the analysis from TCGA (Table S5), indicating that the expression level of *RNF8* had no significant correlation with *CTNNB1* in colon cancer.

Surprisingly, nuclear extraction assays of HCT116 and SW480 cells showed that overexpressing and silencing RNF8 elevated and decreased the protein level of β-catenin in the nuclear fraction, respectively (Figure 3E and 3F, Figure S3E and S3F). Additionally, these phenomena were observed in HEK293 cells with RNF8 overexpression (Figure S3G). Furthermore, immunofluorescence analysis showed that overexpressing and knocking down RNF8 promoted and inhibited β-catenin distribution in nuclei, respectively (Figure 3G and 3H). These results indicated that RNF8 facilitated β-catenin nuclear translocation.

### RNF8 promotes K63 polyubiquitination of β-catenin

**Figure 4:**
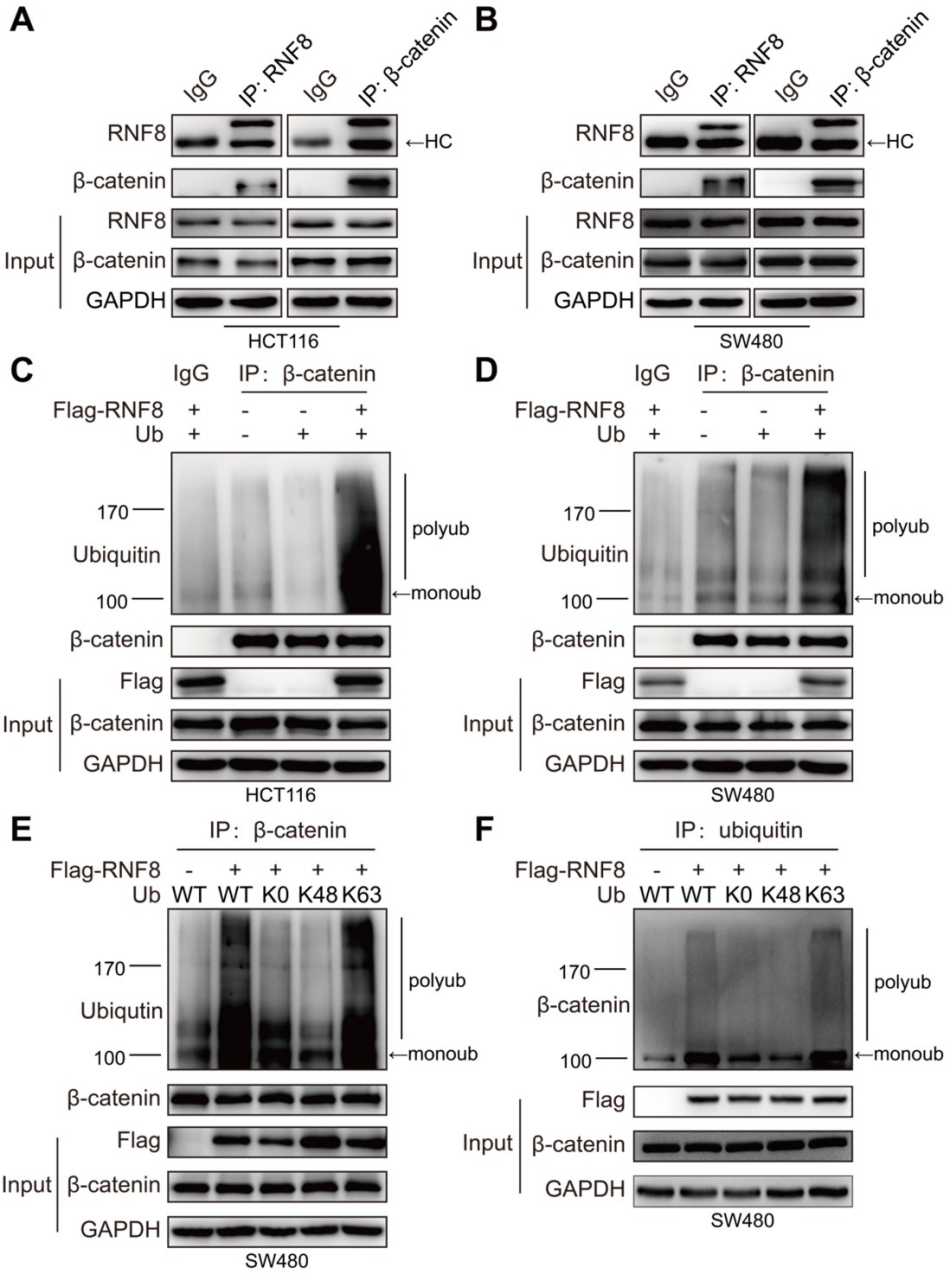
** RNF8 promotes K63 polyubiquitination of β-catenin. (A-B)** RNF8 interacted with β-catenin *in vivo*. Immunoprecipitation was performed with HCT116 and SW480 cell lysates using RNF8 or β-catenin antibodies, and the precipitants were detected by western blotting with the indicated antibodies. HC=heavy chain. **(C-D)** Overexpressing RNF8 induced polyubiquitination of β-catenin. HCT116 and SW480 cells were cotransfected with Flag-RNF8 and a wildtype ubiquitin plasmid, and the cells were subjected to an ubiquitination assays. **(E-F)** RNF8 conjugated K63-linked polyubiquitination chains on β-catenin. SW480 cells were coexpressed with Flag-RNF8 and wildtype ub (WT), or ub without lys (K0), ub with Lys48 (K48) only one lysine residue, ub with Lys63 (K63) only one lysine residue. The cells were then applied to ubiquitination assays.

Several studies have reported that K63 polyubiquitination is necessary for nuclear translocation of proteins such as NRIF, YAP, and IRF5 36-38. It has been reported that β-catenin is transcriptionally activated via K63-linked polyubiquitination by Rad6B 39. However, little is known about whether K63 polyubiquitination regulates β-catenin nuclear translocation. Because RNF8 was reported to add K63 polyubiquitination to substrates such as Twist and TAK1 for their activation 24, 40, we wondered that whether RNF8 could facilitate β-catenin nuclear translocation by assembling K63 polyubiquitination chains. We firstly identified the interaction between RNF8 and β-catenin. We performed immunoprecipitation of whole cell lysates from HCT116 and SW480 cells with the indicated antibodies and detected the relevant proteins in the precipitants by western blotting (Figure 4A and 4B). The results showed that RNF8 interacted with β-catenin *in vivo*.

Subsequently, we examined whether RNF8 promoted the ubiquitination of β-catenin. We overexpressed RNF8 and ubiquitin in HCT116 and SW480 cells and then detected ubiquitination of β-catenin by *in vivo* ubiquitination assay (Figure 4C and 4D). The results demonstrated that overexpression of RNF8 enhanced the polyubiquitination of β-catenin. To further explore RNF8-induced polyubiquitination type of β-catenin, RNF8 together with a wildtype ubiquitin (WT), ubiquitin mutant without lysine (K0) and ubiquitin containing Lys48 (K48) or Lys63 (K63) as the only lysine residue were overexpressed in SW480 cells, followed by ubiquitination assay (Figure 4E and 4F). The results revealed that RNF8 assembled polyubiquitin chains on β-catenin at the K63 ubiquitin linkage, but not K48, indicating that RNF8 primarily promoted K63-linked polyubiquitination of β-catenin.

### RNF8 is required for the proliferation of colon cancer cells

**Figure 5:**
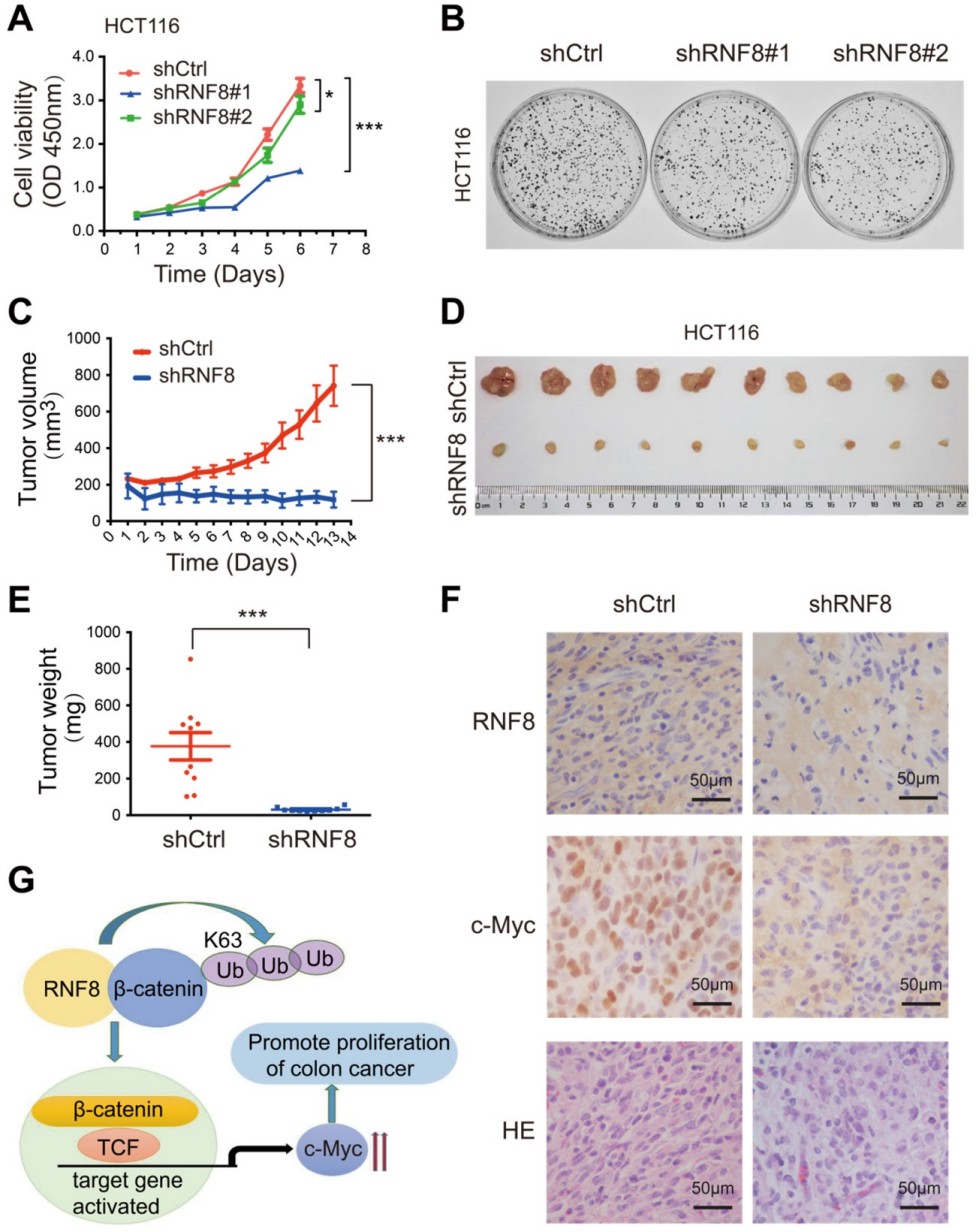
** RNF8 is required for the proliferation of colon cancer cells. (A)** Knockdown of RNF8 inhibited cell growth rate. HCT116 cells with stably knockdown of RNF8 were established and the CCK8 proliferation assay was performed using these cells. *p<0.05; ***p<0.001 (Student's *t*-test) compared with shCtrl control. **(B)** Silencing of RNF8 inhibited the cell growth. Colony formation assay was conducted with stable RNF8 knockdown HCT116 cells. **(C)** Volumes of dissected tumors of stable RNF8 knockdown and control HCT116 cells. ***p<0.001 (Student's *t*-test) compared with shCtrl control. **(D)** Images of xenografted tumors of stable RNF8 knockdown and control HCT116 cells. **(E)** The weight of dissected tumors were calculated and analyzed. ***p<0.001 (Student's *t*-test) compared with shCtrl control. **(F)** Representative images of RNF8 and c-Myc immunohistochemical staining, and HE analysis of the xenografted tumors. **(G)** Schematic representation of RNF8 inducing c-Myc expression by assembling K63 polyubiquitination onto β-catenin and promoting colon cancer cell proliferation.

Recently, RNF8 was reported to participate in breast cancer progression 23-25. In this study, we found that RNF8 promoted c-Myc expression via β-catenin, and the β-catenin-c-Myc pathway is essential for CRC progression 41-43. Thus, we investigated whether RNF8 regulated the proliferation of colon cancer cells. HCT116 and SW480 cells with stable silencing of RNF8 were performed for the CCK8 proliferation assay (Figure 5A, Figure S4A) and colony formation assay (Figure 5B, Figure S4B), which demonstrated that silencing RNF8 retarded the growth of colon cancer cells *in vitro*. However, stable knockdown of RNF8 had no significant effect on the growth of HEK293 cells (Figure S4C), suggesting a specific role of RNF8 in cancer cells.

To further investigate the role of RNF8 in the proliferation of colon cancer cells *in vivo*, we subcutaneously injected stable RNF8 knockdown and control HCT116 cells into 4-week-old female nude mice. The tumor volume was measured every day after injection (Figure 5C), and the results showed that silencing RNF8 significantly reduced the proliferation rate of HCT116 cells *in vivo*. Thirteen days later, the tumors were isolated, photographed (Figure 5D) and weighted (Figure 5E), which showed that RNF8 knockdown tumors were apparently smaller and lighter compared with control tumors. These data demonstrated that knockdown of RNF8 dramatically inhibited the proliferation of colon cancer cells *in vivo*.

Subsequently, we performed immunohistochemistry of the xenograft tumor tissues. The results showed that expression of c-Myc was decreased significantly when RNF8 was knocked down (Figure 5F). These findings were consistent with the above *in vitro* results, suggesting that RNF8 promoted the proliferation of colon cancer cells by upregulation of c-Myc expression.

## Discussion

The ubiquitin E3 ligase RNF8, which primarily functions in transducing DSB signals by conjugating ubiquitin to histones 22, has been reported to participate in tumorigenesis because RNF8 knockout mice develop various tumors spontaneously 44-45, indicating a negative role of RNF8 in tumorigenesis. However, RNF8 appears to have the opposite effect in tumor progression according to recent studies, because it promoted breast cancer proliferation and metastasis by affecting the Wnt/β-catenin pathway and activating the activity of transcription factors such as Twist and ERα 23-25. Consequently, because the role of RNF8 in the progression of other cancers remains unclear, it is important to clarify the function and mechanism of RNF8 in regulating the progression of cancer.

### Positive correlation between RNF8 and c-Myc in colon cancer

According to the global cancer statistics in 2018 1, CRC ranks as third for incidence (10.2%), and it is the second leading cause of death (9.2%) among cancers worldwide. In this study, we detected the transcription level of *RNF8* in colon cancer based on the TCGA. The results showed that *RNF8* mRNA level was significantly higher in colon cancer than in normal tissue (Figure 1A), which prompted us to explore the role of RNF8 in colon cancer progression. Because *MYC* is a well-known oncogene and frequently dysregulated in colon cancer 17, 34, 42 , we analyzed the correlation between *RNF8* and *MYC* using the GEPIA system 27 and analyzing the data from TCGA. We found that *RNF8* had a positive relationship with *MYC* in colon cancer but not in normal tissue (Figure 1C and 1D, Table S1). We then examined RNF8 and c-Myc protein expression patterns in colon cancer tissue compared with benign tissue by immunohistochemistry and confirmed their positive correlation in colon cancer tissues (Figure 1E-H, Table S2). In addition, high expression of RNF8 and c-Myc was related to a poor prognosis of colon cancer patients (Figure 1I and 1J). These results strongly suggested a close relationship between RNF8 and c-Myc in colon cancer progression.

### RNF8 induces c-Myc expression via β-catenin

In addition to its function in DSB signaling transduction, RNF8 was firstly identified as a transcription coactivator of RXRα in 2004 46. Recently, studies have reported that RNF8 acted as a cofactor of transcription factor Twist or ERα in transcriptional regulating of a broad range of genes 24-25. Thus, we hypothesized that RNF8 might regulate the transcription of *MYC*. The results of real-time PCR showed that the transcription level of *MYC* was increased or decreased apparently when RNF8 was overexpressed or knocked down in colon cancer cells (Figure 2A and 2B, Figure S2A and S2B). Moreover, the protein level of c-Myc exhibited similar alterations in colon cancer cells with overexpression or knockdown of RNF8 (Figure 2C and 2D, Figure S2C and S2D).

Since β-catenin is the critical activator for transcription of Wnt/β-catenin pathway target genes 30, we examined whether RNF8-induced c-Myc expression was dependent on β-catenin. The results revealed that the enhancing effect of overexpressed RNF8 on c-Myc expression was compromised by silencing β-catenin in colon cancer cells (Figure 2E and 2F, Figure S2E and S2F), which was suggested that RNF8 promoted c-Myc expression via β-catenin. However, because c-Myc is a transcription regulator 17, whether c-Myc regulates the transcription of RNF8 reciprocally will be explored in our future studies.

### RNF8 facilitates β-catenin nuclear translocation and promotes its polyubiquitination

The Wnt/β-catenin pathway is one of the fundamental signaling cascades in development and homeostasis, and mutations in this pathway lead to hereditary disorders and cancers 33. β-catenin is a critical factor in this pathway and is continually eliminated by the Axin complex in the absence of Wnt. In this process, β-catenin is sequentially phosphorylated by CK1 and GSK3β and subsequently ubiquitinated leading to degradation, which prevents nuclear translocation of β-catenin and the transcription of Wnt target genes 33. When the pathway is activated by Wnt binding to the Frizzled receptor and coreceptor, β-catenin accumulates in the cytoplasm and then translocates into the nucleus, activating Wnt target genes transcription 33. Constitutively activated β-catenin signaling is an essential mechanism for the oncogenesis and progression of CRC 47. Hence, we detected the phosphorylation and protein levels of β-catenin in colon cancer cells with RNF8 overexpression and silencing. Unfortunately, we found no obvious changes in these cells (Figure 3A-D, Figure S3A-D), which was in accordance with the analysis of the correlation of *RNF8* and *CTNNB1* mRNA expression from TCGA (Table S5). However, this is inconsistent with a previous report of breast cancer 23, which may be due to different cancer cell lines.

Nevertheless, we observed a significant increase and decrease of β-catenin nuclear translocation when RNF8 was overexpressed and knocked down in colon cancer cells, respectively (Figure 3E and 3F, Figure S3E and S3F). RNF8 facilitating β-catenin nuclear distribution was also found by immunofluorescence analysis (Figure 3G and 3H). However, the effect of RNF8 on β-catenin nuclear translocation was also observed in the nuclear extraction assay of human embryonic kidney HEK293 cells (Figure S3G), suggesting that RNF8 facilitating β-catenin nuclear translocation was not restricted to colon cancer and requires further investigation in other cell types.

Our results encouraged us to explore how RNF8 regulated β-catenin nuclear translocation. As a result, we found an interaction between RNF8 and β-catenin in colon cancer cells *in vivo* (Figure 4A and 4B). Because RNF8 is an ubiquitin E3 ligase, we next examined whether the ubiquitination level of β-catenin was influenced by RNF8. In ubiquitination assay, we observed that overexpression of RNF8 dramatically elevated K63-linked polyubiquitination of β-catenin in colon cancer cells (Figure 4C-F). It has been reported by several studies that K63 polyubiquitination is a positive modification for nuclear translocation of proteins such as Twist, NRIF, YAP and IRF5 24, 36-38. Thus, the enhancing effect of RNF8 on K63 polyubiquitination of β-catenin might be a potential mechanism to induce its nuclear translocation.

### RNF8 is required for the proliferation of colon cancer cells

Since our results showed that RNF8 upregulated c-Myc expression by facilitating β-catenin nuclear translocation through K63-linked polyubiquitination, we next examined whether RNF8 affected the proliferation of colon cancer cells. CCK8 proliferation and colony formation assays showed that silencing RNF8 significantly inhibited the growth of colon cancer cells *in vitro*, but not that of HEK293 cells (Figure 5A and 5B, Figure S4A-C). It was suggested a specific role of RNF8 in cancer cells. Moreover, knockdown of RNF8 obviously retarded the proliferation of HCT116 xenografts *in vivo* (Figure 5C-E) and reduced the expression of c-Myc in xenograft tissues (Figure 5F).

Based on the above results, we suggested that RNF8 assembled K63-linked polyubiquitination on β-catenin and facilitated its nuclear translocation in colon cancer cells, which in turn enhanced the expression of Wnt/β-catenin target gene *MYC*, promoting the proliferation of colon cancer cells (Figure 5G). These findings revealed a new mechanism for the progression of colon cancer, which might be a potential therapeutic target for the treatment of colon cancer.

## Supplementary Material

Supplementary figures and tables.
