# Supplementary material for: RNF8 induces β-catenin-mediated c-Myc expression and promotes colon cancer proliferation
Source: Int J Biol Sci. 2020 May 1;16(12):2051–62. doi: 10.7150/ijbs.44119 (PMC7294952; doi:10.7150/ijbs.44119)
Supplement: Supplementary file 1 — Supplementary figures and tables. [file ijbsv16p2051s1.pdf]

Supplementary Materials

Materials and Methods

Cell culture

HEK293T, HEK293, HCT116, and SW480 cells were obtained from the Cell Bank of Chinese Academy of Sciences (Shanghai, China). HEK293T and HEK293 cells were cultured in Dulbecco's modified Eagle's medium (Biological Industries, 01-055-1A). HCT116 cells were cultured in Iscove's modified Dulbecco's medium (Gibco, C12440500BT) and SW480 cells were cultured in RPMI 1640 medium (Biological Industries, 01-100-1A). All culture media were supplemented with 10% fetal bovine serum (FBS; Gibco, 10099141) and penicillin (100 U/ml)/streptomycin (100 μg/ml) (Gibco, 15140122). All cells were cultured in a humidified incubator at 37°C containing 5% CO_2.

Antibodies

Antibodies against the following molecules were used in this study: RNF8 (Santa Cruz Biotechnology, SC-271462), c-Myc (Proteintech, Cat. No. 10828-1-AP), β-catenin (Santa Cruz Biotechnology, SC-7963), α-tubulin (Sigma, T6199), β-actin (Sigma, A1978), GAPDH (Cell Signaling Technology, 97166T), Flag (Cell Signaling Technology, 2368s), ubiquitin (Cell Signaling Technology, 3933), phospho-β-catenin (Ser33/Ser37/Thr41) (Cell Signaling Technology, 9561), and phospho-β-catenin (Ser45) (Cell Signaling Technology, 9564). Horseradish peroxidase (HRP) goat

anti-rabbit and anti-mouse IgG (ABclonal Biotech, Co., Ltd., AS003) was used as a secondary antibody.

Plasmids, siRNA, and transfection

The Flag-RNF8 expression and wildtype ubiquitin plasmids were gifts from the Laboratory of Chromatin Biology of China Medical University [1], and pRK5-HA-UB (K0), pRK5-HA-UB (K48), and pRK5-HA-UB (K63) were donated by Ronggui Hu's group at the Institute of Biochemistry and Cell Biology, Shanghai Institutes for Biological Sciences, Chinese Academy of Sciences [2]. Control siRNA (siCtrl) and siRNA against RNF8 (siRNF8) were purchased from Sigma (Shanghai, China). Control siRNA (siCtrl) and siRNA against β-catenin (siβ-catenin) were purchased from Ribobio (Shanghai, China). HCT116 and SW480 cells were transiently transfected with expression plasmids and siRNA using jetPRIME transfection reagent (Polyplus transfection, 114-15) or Lipofectamine 3000 transfection reagent (Thermo Fisher Scientific, L3000015), according to the manufacturers' instructions. Cells were harvested for subsequent experiments after 48 hours.

Western blot analysis

Cell lysates were separated by sodium dodecyl sulfate-polyacrylamide gel electrophoresis and then transferred to a polyvinylidene difluoride membrane (Millipore, IPVH00010). After blocking with 5% dry milk, the membranes were

incubated with the recommended dilution of the antibody at 4°C overnight, washed three times with TBS-T, and then incubated with the HRP-labeled secondary antibody. After washing with TBS-T three times, chemiluminescence detection was performed with ECL reagent (Tanon Science & Technology Co., Ltd., Shanghai, China).

Immunoprecipitation assay

For immunoprecipitation, HCT116 and SW480 cells were lysated and equal amounts of proteins after preclearing were immunoprecipitated with antibodies at 4°C for 3 hours, followed by addition of protein A/G coupled sepharose beads (Santa Cruz Biotechnology, sc-2003) at 4°C overnight. The protein-antibody complexes were washed with cold PBS three times at 4°C and eluted with loading buffer by boiling for 10 minutes. The proteins were subjected to western blot analysis as described above.

Immunofluorescence

HCT116 cells transfected with Flag-RNF8 for 24 hours and stable RNF8 knockdown HCT116 cells were seeded in 12-well culture plates containing gelatin-coated coverslips and maintained in Iscove's modified Dulbecco's medium with 10% FBS for 24 hours. Then, the cells were fixed with 4% paraformaldehyde and permeabilized with 0.1% Triton X-100. Cells were incubated with an anti-β-catenin antibody, followed by washing in PBS three times, and then incubated with an Alexa Fluor 488 secondary antibody (Thermo Fisher Scientific). Nuclei were

counterstained with DAPI (Sigma). Immunofluorescence staining was visualized using the Zeiss confocal laser scanning system 510.

Statistical analysis

SPSS 17.0 (SPSS, Inc, Chicago IL, USA) was used for statistical analysis. $p<0.05$ was considered as statistically significant. The non-parametric t-test for categorical data and the two-tailed Student's *t*-test for continuous variables (expressed as the mean ± SD) were used to evaluate the differences between experimental and control groups. Spearman's correlation was used to analyze the relationship of RNF8 and c-Myc expression with clinicopathological parameters. A log-rank test of the Kaplan-Meier curve was conducted to evaluate the relationship between RNF8 or c-Myc expression and survival of colon cancer patients.

References

1. Wang S, Luo H, Wang C, Sun H, Sun G, Sun N, et al. RNF8 identified as a co-activator of estrogen receptor alpha promotes cell growth in breast cancer. Biochim Biophys Acta Mol Basis Dis. 2017; 1863: 1615-28.
2. Liu Z, Chen P, Gao H, Gu Y, Yang J, Peng H, et al. Ubiquitylation of autophagy receptor Optineurin by HACE1 activates selective autophagy for tumor suppression. Cancer Cell. 2014; 26: 106-20.

Supplementary Figures

Figure S1

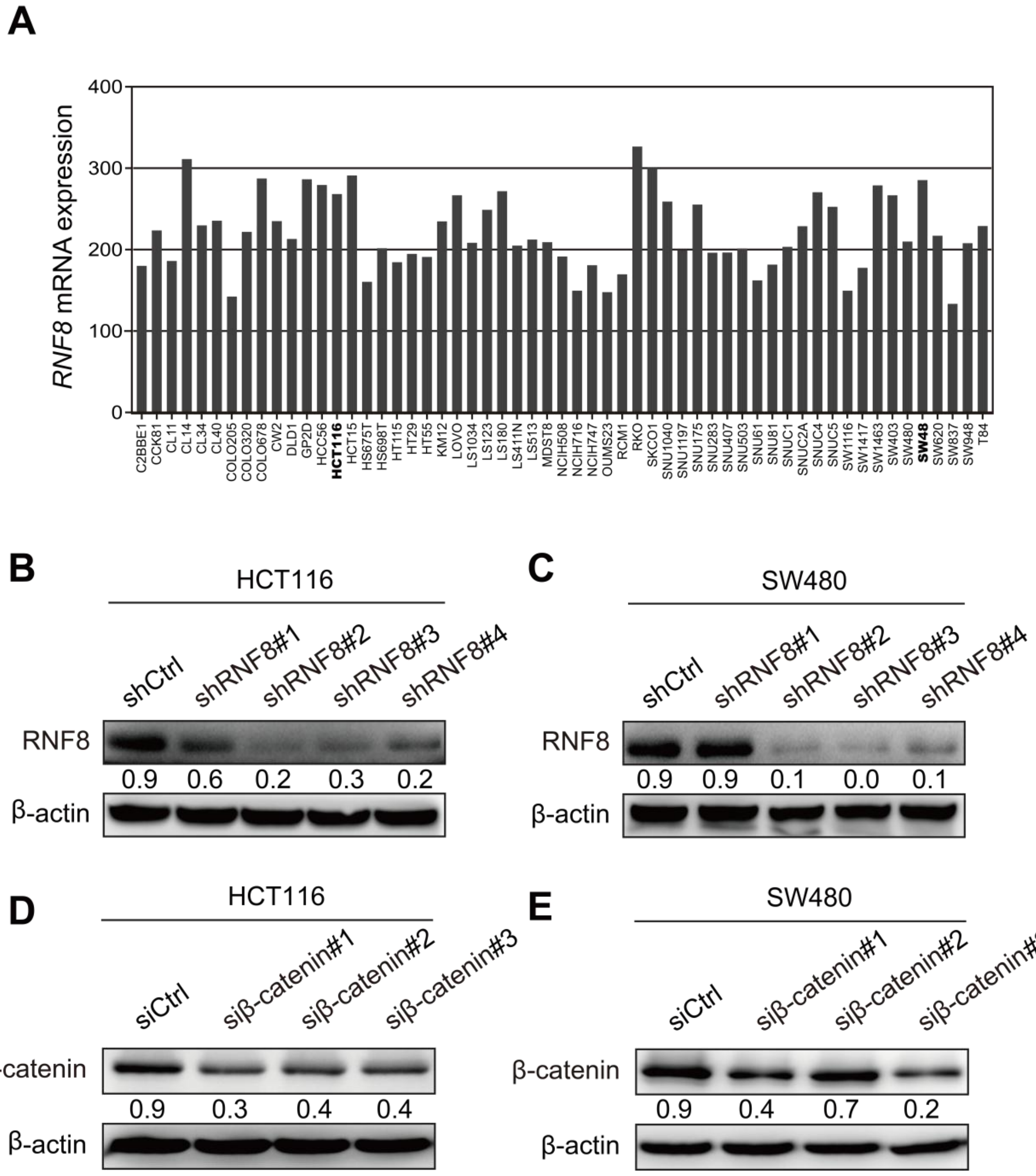

Figure S1. **(A)** Analysis of *RNF8* transcription in colon cancer cell lines based on the Cancer Cell Line Encyclopedia. **(B-C)** Knockdown efficiency of shRNA targeting RNF8 in HCT116 and SW480 cells. **(D-E)** Silencing efficiency of siRNA targeting β-catenin in HCT116 and SW480 cells.

Figure S2

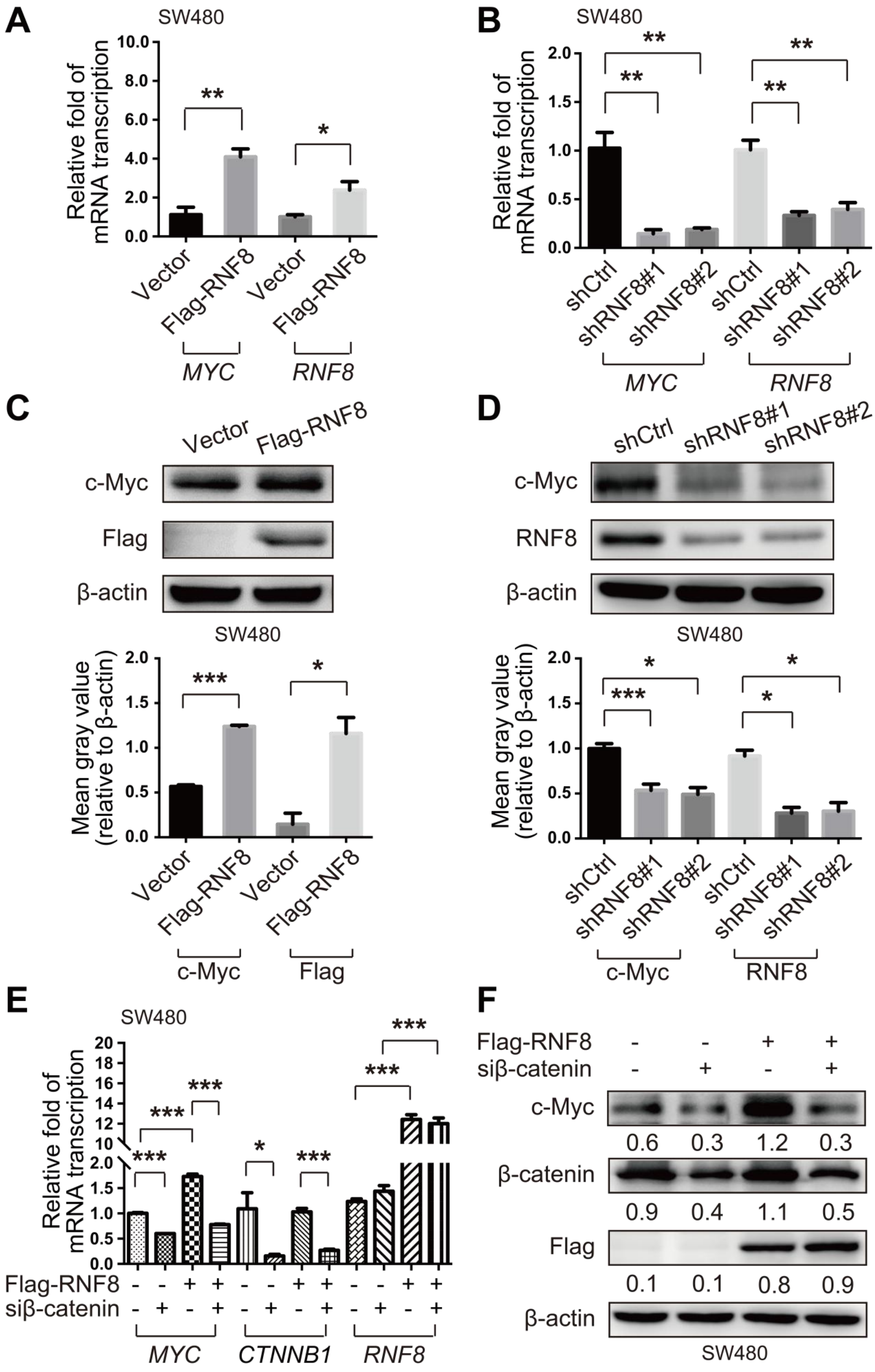
A
SW480
Relative fold of mRNA transcription
10.0
8.0
6.0
4.0
2.0
0.0
**
*
Vector
Flag-RNF8
Vector
Flag-RNF8
MYC
RNF8
B
SW480
Relative fold of mRNA transcription
2.0
1.5
1.0
0.5
0.0
**
**
**
**
shCtrl
shRNF8#1
shRNF8#2
shCtrl
shRNF8#1
shRNF8#2
MYC
RNF8
C
Vector
Flag-RNF8
c-Myc
Flag
β-actin
SW480
Mean gray value (relative to β-actin)
2.0
1.5
1.0
0.5
0.0
***
*
Vector
Flag-RNF8
Vector
Flag-RNF8
c-Myc
Flag
D
shCtrl
shRNF8#1
shRNF8#2
c-Myc
RNF8
β-actin
SW480
Mean gray value (relative to β-actin)
2.0
1.5
1.0
0.5
0.0
*
***
*
*
shCtrl
shRNF8#1
shRNF8#2
shCtrl
shRNF8#1
shRNF8#2
c-Myc
RNF8
E
SW480
Relative fold of mRNA transcription
20
18
16
14
12
10
2.0
1.5
1.0
0.5
0.0
***
***
***
*
***
***
***
Flag-RNF8 - - + + - - + + - - + +
siβ-catenin - + - + - + - + - + - +
MYC
CTNNB1
RNF8
F
Flag-RNF8 - - + +
siβ-catenin - + - +
c-Myc
0.6 0.3 1.2 0.3
β-catenin
0.9 0.4 1.1 0.5
Flag
0.1 0.1 0.8 0.9
β-actin
SW480

Figure S2. RNF8 induces c-Myc expression via β-catenin in SW480 cells. (A-B) Overexpression and knockdown of RNF8 increased and decreased *MYC* gene transcription in SW480 cells, respectively. *$p<0.05$; **$p<0.01$ (Student's *t*-test) compared with the vector and shCtrl controls. **(C-D)** Overexpression and knockdown of RNF8 increased and decreased the c-Myc protein level in SW480 cells, respectively. Gray value analyses are shown below western blot images. *$p<0.05$; ***$p<0.001$ (Student's *t*-test). **(E-F)** Silencing β-catenin suppressed the enhancing effect of RNF8 overexpression on c-Myc mRNA and protein expression in SW480 cells. *$p<0.05$; ***$p<0.001$ (Student's *t*-test).

Figure S3

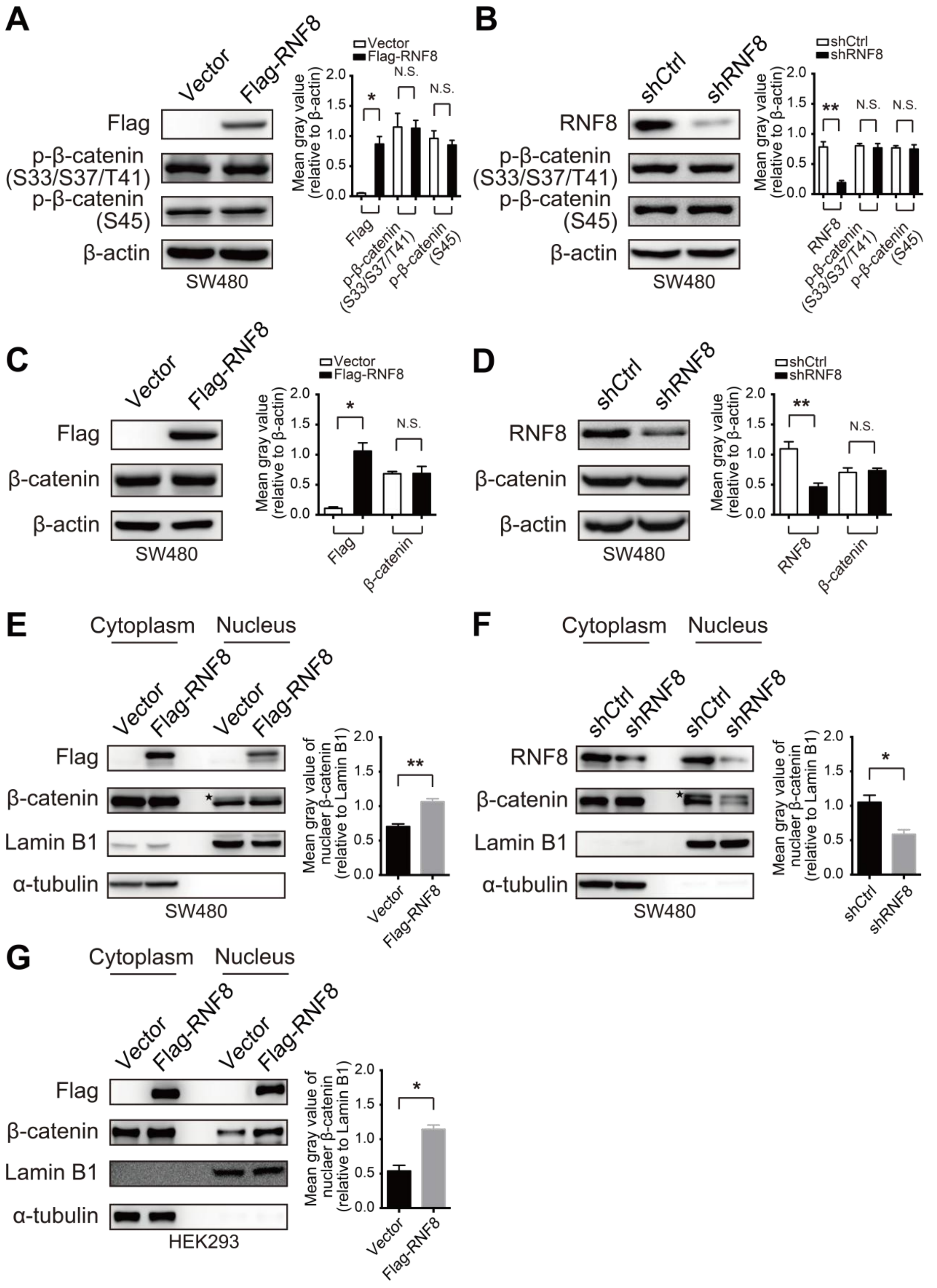

Figure S3. RNF8 facilitates β-catenin nuclear translocation in SW480 cells. (A-B) Overexpression or knockdown of RNF8 hardly influenced the phosphorylation level of β-catenin at S33/S37/T41 and S45 in SW480 cells. Gray value analyses are shown on the right. *p<0.05; **p<0.01; N.S.=no significant difference (Student's *t*-test). **(C-D)** The protein level of β-catenin showed little alteration when RNF8 was overexpressed or knocked down in SW480 cells. Gray

value analyses are shown on the right. *$p<0.05$; **$p<0.01$; N.S.=no significant difference (Student's *t*-test). **(E-F)** Overexpression and silencing of RNF8 increased and reduced nuclear translocation of β-catenin in SW480 cells, respectively. Asterisk indicates a non-specific band. Gray value analyses of β-catenin in the nuclear fraction are shown on the right. *$p<0.05$; **$p<0.01$ (Student's *t*-test). **(G)** Overexpression of RNF8 promoted β-catenin nuclear accumulation in HEK293 cells. Gray value analysis of β-catenin in the nuclear fraction is shown on the right. *$p<0.05$ (Student's *t*-test).

Figure S4

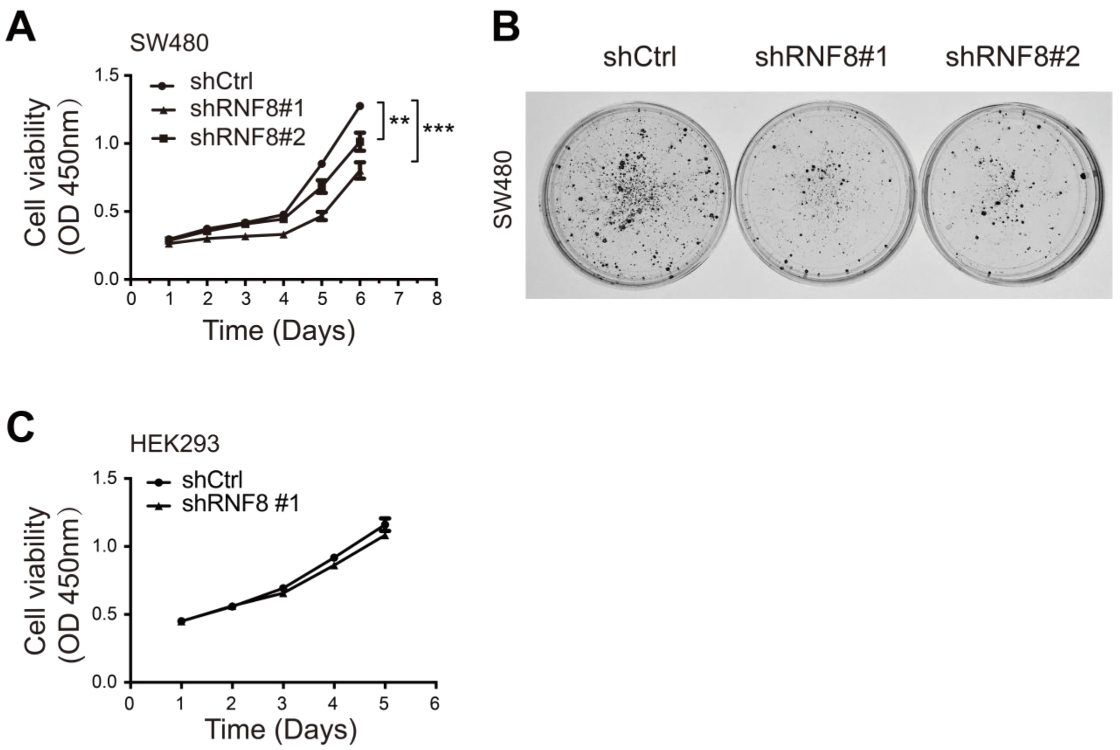

Figure S4. RNF8 is required for the proliferation of SW480 colon cancer cells. (A-B) Stable knockdown of RNF8 inhibited the growth of SW480 cells examined by CCK8 proliferation and colony formation assays. **p<0.01; ***p<0.001 (Student's *t*-test) compared with shCtrl control. **(C)** Silencing RNF8 had no significant effect on the growth rate of HEK293 cells as determined by CCK8 proliferation assay.

Supplementary Tables

Table S1: The relationship between *RNF8* and *MYC* mRNA levels in colon cancer tissues and normal tissues in TCGA database.

	MYC			
	normal (N=41)		cancer (N=478)	
	r	p	r	p
RNF8	-0.057	0.725	**0.217****	**0.000**

**: Significant correlation (Both sides) at 0.01 level

Table S2: The correlation between RNF8 and c-Myc protein levels in colon cancer tissues and benign tissues.

	**c-Myc**			
	normal (N=78)		cancer (N=99)	
	r	p	r	p
**RNF8**	0.210	0.065	**0.299****	**0.003**

**: Significant correlation (both sides) at 0.01 level

Table S3: Colon cancer patients with high RNF8 or c-Myc expression suffered high risk of death.

	HR	95%CI		p
		low	high	
**RNF8**	2.101	1.210	3.648	0.008
**c-Myc**	1.769	1.015	3.082	0.044

Table S4: RNF8 and c-Myc staining and clinicopathological characteristics of colon cancer patients.

characteristics	**RNF8** (99)				**c-Myc** (99)			
	High (%)	Low (%)	total	p	High (%)	Low (%)	total	p
**age**								
＞65	17(31.5)	37(68.5)	54	0.405	19(35.2)	35(64.8)	55	1.000
≤65	18(40.0)	27(60.0)	45		16(35.6)	29(64.4)	45	
**sex**								
male	18(36.7)	31(63.3)	49	0.835	19(38.8)	30(61.2)	49	0.532
female	17(34.0)	33(66.0)	50		16(32.0)	34(68.0)	50	
**TNM stage**								
I + II	20(33.3)	40(66.7)	60	0.669	17(28.3)	43(71.7)	60	0.087
III + IV	15(38.5)	24(61.5)	39		18(46.2)	21(53.8)	39	
**Lymph node metastasis**								
positive	15(38.5)	24(61.5)	39	0.515	18(46.2)	21(53.8)	39	0.05
negative	18(31.0)	40 (69.0)	58		15(25.9)	43(74.1)	58	

Table S5: The relationship between *RNF8* and *CTNNB1* mRNA level in colon cancer tissues and normal tissues in TCGA database.

	CTNNB1			
	normal (N=41)		cancer (N=478)	
	r	p	r	p
RNF8	-0.293	0.063	0.038	0.407

Table S6: Quantitative real-time PCR primers for genes as indicated.

Name	Sense (5'-3')	Anti-sense (5'-3')
RNF8	CAGGCAAGAGTGGAGCAACTA	TCCATTAGAGCCCAATGCTCC
MYC	GTCAAGAGGCGAACACACAAC	TTGGACGGACAGGATGTATGC
CTNNB1	AAAGCGGCTGTTAGTCACTGG	CGAGTCATTGCATACTGTCCAT
GAPDH	GGAGCGAGATCCCTCCAAAAT	GGCTGTTGTCATACTTCTCATGG
